# Late-Onset Antibody Deficiency Due to Monoallelic Alterations in *NFKB1*

**DOI:** 10.3389/fimmu.2019.02618

**Published:** 2019-11-14

**Authors:** Claudia Schröder, Georgios Sogkas, Manfred Fliegauf, Thilo Dörk, Di Liu, Leif G. Hanitsch, Sophie Steiner, Carmen Scheibenbogen, Roland Jacobs, Bodo Grimbacher, Reinhold E. Schmidt, Faranaz Atschekzei

**Affiliations:** ^1^Department for Clinical Immunology and Rheumatology, Hannover Medical School, Hanover, Germany; ^2^Hannover Biomedical Research School (HBRS), Hannover Medical School, Hanover, Germany; ^3^Center for Chronic Immunodeficiency, Center for Pediatrics and Adolescent Medicine, University Medical Center Freiburg, Freiburg im Breisgau, Germany; ^4^CIBSS – Centre for Integrative Biological Signalling Studies, Albert-Ludwigs University, Freiburg, Germany; ^5^Gynaecology Research Unit, Hannover Medical School, Hanover, Germany; ^6^Institute for Medical Immunology, Charité University Medicine Berlin, Berlin, Germany; ^7^DZIF – German Center for Infection Research, Hannover Medical School, Hanover, Germany; ^8^RESIST – Cluster of Excellence 2155 to Hanover Medical School, Satellite Center Freiburg, Hanover, Germany

**Keywords:** nuclear factor kappa B subunit 1, combined immunodeficiency, common variable immunodeficiency, *NFKB1* haploinsufficiency, hypogammaglobulinemia, late-onset antibody deficiency

## Abstract

Adult-onset primary immunodeficiency is characterized by recurrent infections, hypogammaglobulinemia, and poor antibody response to vaccines. In this study, we have analyzed targeted gene panel sequencing results of 270 patients diagnosed with antibody deficiency and identified five disease-associated variants in *NFKB1* in five unrelated families. We detected two single base pair deletions and two single base pair insertions, causing severe protein truncations, and one missense mutation. Immunoblotting, lymphocyte stimulation, immunophenotyping, and ectopic expression assays demonstrated the functional relevance of *NFKB1* mutations. Besides antibody deficiency, clinical manifestations included infections, autoimmune features, lymphoproliferation, lymphoma, Addison's disease, type 2 diabetes and asthma. Although partial clinical penetrance was observed in almost all pedigrees, all carriers presented a deficiency in certain serum immunoglobulins and the majority showed a lack of memory B cells (CD19^+^CD27^+^). Among all tested genes, *NFKB1* alterations were the most common monoallelic cause of antibody deficiency in our cohort.

## Introduction

Human primary immunodeficiency (PID) comprises a group of 330 distinct disorders with 354 monogenic inborn errors of immunity (1). Patients present considerable clinical and genetic heterogeneity. The most common manifestations are infections, allergy, autoimmunity, malignancy, and auto-inflammation (2). The first manifestation usually erupts in newborns or early childhood. However, the frequency of diagnosed adult-onset PIDs increases (3). The majority of cases are diagnosed predominantly with humoral deficiencies and half of these display late disease onset. The most common symptomatic immunodeficiency, which typically manifests in adulthood is CVID (4). So far, several single-gene defects such as *ICOS, CD19, CD20, CD21, CD27, CD81, IL21, IL21R, LRBA, PRKCD, RAC2, TNFSF12, CTLA4, PLCG2, NFKB1, NFKB2, PIK3CD, PIK3R1, VAV1, BLK, IKZF1, IRF2BP2*, as well as mutations in *TNFRSF13B* and *TNFRSF13C* have been identified in CVID (1, 2, 5, 6). This led to founding of new monogenic PID disorders out of erstwhile CVID phenotypes. So far, deleterious variants in nuclear factor kappa B subunit 1 (*NFKB1*) gene appear to be the most common cause in Europeans (7) followed by variants in *PIK3CD, LRBA* and *CTLA4* (5). However, monogenic forms account for only 10% of CVID patients (8, 9). Further studies have shown that several variants in these genes are associated with incomplete disease penetrance (10–12). This implies that further factors such as modifier genes and/or environmental impacts might play a crucial role in disease onset and course (13).

*NFKB1* encodes for the transcription factor NF-κB1, which is expressed in virtually all cell types (14). It is implicated in cell proliferation and survival, immune response, and inflammation (15). The NF-κB transcription factor family comprises of seven proteins: NF-κB1 (p105/p50), NF-κB 2 (p102/p52), RelA, RelB, and c-Rel (16, 17). All members share N-terminally the Rel homology domain (RHD), which is important for DNA-binding, dimerization and interaction with inhibitory proteins (14). Additionally, RelA, RelB and c-Rel contain a C-terminal transactivation domain and NF-κB1 or NF-κB2 contains ankyrin repeats (16). Both, NF-κB1 and NF-κB2 are expressed as “inactive” precursor proteins (p105 and p100) and undergo partial proteolytic procession of their C-terminal half to generate the active nuclear transcription factor subunits p50 and p52, respectively. NF-κB proteins can form various homo- or heterodimers with unique and distinct functions. For instance, p50/p52 homodimers act as transcriptional repressor, since they lack transactivation domain. In contrast, heterodimers of p50 or p52 with one of the Rel proteins act as transcriptional activators or inflammatory response inducers. The most abundant heterodimers are p50/RelA and p52/RelB.

Two signaling pathways have been described: the classical or canonical NF-κB pathway involving NF-κB1 and the non-canonical pathway involving NF-κB2 (18). In the classical pathway p105 is constitutively processed to p50 and predominantly assembles with RelA. This complex kept inactive in the cytoplasm through binding of the inhibitory IκBα protein. Upon stimulation, IκBα is phosphorylated, polyubiquitinated and degraded by the 26S proteasome. Subsequently the p50/RelA heterodimer translocates to the nucleus and binds to kappa-B sites in the regulatory regions of their target genes. In contrast to the classical pathway, the NF-κB2 precursor p100 is processed to its transcriptional active subunit p52 only after activation of specific receptors on B cells such as BAFFR or CD40 and is therefore mainly involved in B-cell function. Functional p52 haploinsufficiency has been associated with CVID (19, 20). Inappropriate regulation of NF-κB has been linked to a variety of diseases, including asthma, allergy, inflammatory bowel disease, systemic lupus erythematosus, leukemia, and lymphomas (18, 21, 22). More recently, it has been shown that mutations in *NFKB1* that lead to p50 haploinsufficiency result in phenotypes falling under CVID (11).

*NFKB1* variants can be divided into four main groups: (1) haploinsufficiency mutations such as nonsense or frameshift mutations in the N-terminal part, typically cause severely truncated, non-functional proteins which probably undergo rapid decay; (2) “precursor skipping mutations” leading to truncation in the central part of p105 and cause expression of p50-like mutant proteins; (3) missense mutations in the N-terminal half, affecting both p105 and p50 and (4) missense variants in the C-terminal part, affecting only p105 (23).

In this work, we report seven adult-onset PID cases due to *NFKB1* mutations, including four novel deleterious variants. Having analyzed sequencing panel data from 270 patients with primary immunodeficiency falling under CVID/late-onset CID, we conclude that *NFKB1* alterations are the most common monoallelic cause of antibody deficiency in our cohort.

## Materials and Methods

The study was approved by the institutional medical ethical committee at Hannover Medical School (ethics approval number: 5582). The written consent of all study participants was obtained.

### Isolation of Genomic DNA and Sequencing Methods

Genomic DNA (gDNA) was isolated from peripheral blood of patients and healthy donors with QIAamp Kit (*Qiagen*). For mutational analysis targeted resequencing was performed as previously described (24), using *Agilent Technologies*. Therefore, we used a gene panel comprised of known and candidate genes associated with primary immunodeficiencies (Supplementary Table 1). Next generation sequencing was performed on MiSeq desktop sequencer (*Illumina*). Variants were annotated with *Agilent SureCall* software (*Agilent Technologies*) and verified by Sanger sequencing of genomic PCR products (*Eurofins Genomics*). Primers in Supplementary Table 2 (*Eurogentec*) were used for PCR and Sanger sequencing according to standard techniques.

### Cloning

The cDNAs encoding mutant p105 and p50 were purchased from GeneArt Gene Synthesis (*Invitrogen*) or generated by site-directed mutagenesis and subcloned into the pEGFP-C1 expression vector (*Takara/Clontech*) to generate N-terminally EGFP-tagged constructs. Competent *E.coli* (*NEB*, 5-alpha) were transformed with the vectors constructs and plasmids were isolated with NucleoSpin Plasmid Kit (*Macherey-Nagel*).

### Cell Culture and Transfection

Peripheral blood mononuclear cells (PBMCs) were isolated by density gradient centrifugation over Ficoll. Cells were cultured in R10 medium composed of RPMI 1640 Medium, supplemented with 1% penicillin-streptomycin, 1 mM L-Glutamin and 10% FCS (all from *Merck*).

HEK293T cells were grown in Dulbecco's modified Eagle's medium (*Lonza*) supplemented with 10% FCS (*Merck*) and 1% penicillin-streptomycin (*Merck*). Transient transfection of constructs into HEK293T cells were carried out using X-tremeGENE HP DNA Transfection Reagent (*Roche*) according to the suppliers' recommendations.

### Lymphocyte Stimulation Assays

For stimulation experiments, phorbol 12-myristate 13-acetate (PMA; 50 ng/ml) and ionomycin (1 μg/ml) were added as indicated. After incubation cells were harvested and frozen until further processing.

For standard T cell proliferation assays, PBMCs were stimulated with phytohemagglutinin (PHA), concanavalin A (ConA), pokeweed mitogen (PWM), purified protein derivative (PPD), interleukin 2 (IL-2) and anti-CD3mAb as described previously (25).

### Fluorescence Staining and Confocal Imaging

Transiently transfected HEK293T cells expressing EGFP alone or N-terminally EGFP-tagged p50-WT, p105-WT, p50-R157P, p105-R157P, p.I567Nfs^*^6, or p.S338Lfs^*^94 were fixed with 4% paraformaldehyde (PFA) and nuclei were stained with DAPI (*Carl Roth*). For microscopy, glass coverslips were mounted onto glass slides with fluorescencemounting medium (*Dako*). Confocal fluorescence images were taken on an Olympus FV 1000 laser scanning microscope. Images were evaluated and processed with Fiji software.

### Protein Purification

Proteins from PBMCs were isolated with the NucleoSpin RNA/Protein Kit (*Macherey-Nagel*). Nuclear and cytoplasmic proteins from transfected HEK293T cells were obtained with the NE-PER Nuclear and Cytoplasmic Extraction Reagents (*Thermo Scientific*). Concentrations were measured with Protein Quantification Kit (*Macherey-Nagel*).

### Western Blotting

Proteins were separated on 8% GoPAGE Bis-Tris gels (*7bioscience*) and transferred onto Invitrolon PVDF membranes (*Thermo Scientific*). A primary polyclonal rabbit antibody directed against the N-terminal amino acids was used to detect both p105 and p50 (#3035; *Cell Signaling*). A monoclonal rabbit antibody was used to detect levels of phosphorylated p105 at serine 933 (#4806; *Cell Signaling*). Horseradish-peroxidase-coupled goat anti-rabbit secondary antibody (#6721; abcam) was used to detect signals via enhanced chemiluminescence (SuperSignal West Dura Extended Duration Substrate; *Thermo Fischer*). For loading controls mouse antibody to GAPDH (#97166, *Cell Signaling*) and anti-mouse IgG (#7076, *Cell Signaling*), or α-tubulin (#4074; *abcam*), and anti-rabbit secondary antibody (#6721; *abcam*) or mouse antibody to beta actin (#197277; *abcam*) directly coupled to horseradish-peroxidase was used. Densitometric analyses of western blots were performed with ImageJ software (Version 1.52 g) and the graphs were prepared using GraphPad Prism 5.

### Phenotypic Analyses

Phenotypic analyses were performed with multicolor immunofluorescences staining of PBMCs utilizing directly labeled mAb as described previously (26, 27). Samples were analyzed using a FACSCanto II flow cytometer with Cell Quest software (Becton Dickinson). Offline data analysis was performed by using FCS Express software V6 (Denovo Software).

### Amino-Acid Interaction Analysis

A three-dimensional template for the structure of NF-κB p50 dimer bound to DNA was downloaded from the worldwide Protein Data Bank (http://www.wwpdb.org/; PDB code 1SVC), and was analyzed in detail for direct interactions between the single residue Arg157 and all other residues of the protein using the PyMOL Molecular Graphics System, Version 2.0 Schrödinger, LLC (https://www.pymol.org/2/). The interaction network change after replacement of residue Arg157 with residue Pro157 was analyzed using the “Mutagenesis” function of PyMOL.

## Results

### *NFKB1* Mutations Cause Late-Onset Primary Immunodeficiency

We studied nine individuals from five unrelated kindred with monoallelic *NFKB1* mutations (Table 1). The genetic defect either results in p50 haploinsufficiency or predicts the expression of p50-like proteins or cause a single amino acid change (Table 2). All affected individuals were alive at time of reporting, after a mean follow-up period of 8.9 years (median follow-up period: 4 years). One subject (S7) has been lost from the follow-up.

**Table 1:** Clinical characteristics of subjects with *NFKB1* mutations.

**Family** **ID**	**Subject** **ID**	**Current age (years old)**	**Age at first manifestation**	**Age at first diagnosis of hypogamma-globulinemia**	**Infections**	**Autoimmune manifestations**	**Lymphoproliferation**	**Malignancy**	**Other**
									
Fam A	S1	35	16	18	Recur. bronchitis and sinusitis	Adult-onset Still's disease, AIHA	Lymphadenopathy		
									
Fam B	S2	61	35	38	Recur. bronchitis, sinutitis, CMV-colitis (during R-CHOP)		Splenomegaly, nodular lymphoid hyperplasia of the gastrointestinal tract, nodular regenerative hyperplasia of the liver	Non-Hodgkin lymphoma	Addison's disease, diabetes mellitus type 2
									
	S3	33	n.a.	n.a.					Asthma
	S4	24	n.a.	18					Asthma
Fam C	S5	56	50	52	Recur. bronchitis/ sinusitis				
	S6	50	37	40	Recur. gastrointestinal infections (*S. enteritis*), recur. warts, recur. bronchitis/ sinusitis				
Fam D	S7	61	42	53	Recur. pneumonias, recur. bronchitis/ sinusitis,	ITP	Lymphadenopathy, splenomegaly		
					Generalized shingles				
Fam E	S8	45	41	42	Pneumonia, otitis media, recur. bronchitis				
	S9	42	37	39	Recur. bronchitis, sinusitis. Pneumonia and sepsis through *S. pnemoniae*				

*n.a., not available; AIHA, autoimmune hemolytic anemia; ITP, idiopathic thrombocytopenic purpura; recur, recurrent; CMV, cytomegalovirus; R-CHOP, rituximab, cyclophosphamide, hydroxydaunorubicin, oncovin, prednisolone*.

**Table 2:** Immunological investigations of subjects with *NFKB1* alterations.

	**Haploinsufficiency mutation**						**Precorsor-skipping mutation**	**Missense mutation**		**Normal range/** **control value**
	**Fam A**	**Fam B**			**Fam C**		**Fam D**	**Fam E**		
**Subject ID**	**S1**	**S2**	**S3**	**S4**	**S5**	**S6**	**S7**	**S8**	**S9**	
**Immunoglobulins: year**	2017	2013	2018	2018	2015	2009	2011	2016	2016	
IgG (g/L)	***2.11***	***4.38***	10.18	***6.6***	***6.05***	***0.07***	***2.14***	***<0.33***	***0.45***	7–16
IgG1 (g/L)	***1.39***	***3.30***	8.35	5.00	***4.85***	***0.04***	***1.66***	***<0.22***	***0.35***	4.9–11.4
IgG2 (g/L)	***0.68***	***1.20***	***1.34***	1.72	***0.61***	***0.09***	***0.14***	***<0.003***	***0.09***	1.5–6.4
IgG3 (g/L)	***0.07***	***0.07***	0.28	0.35	***0.17***	***0.01***	0.35	***<0.11***	***0.02***	0.2–1.1
IgG4 (g/L)	***0.04***	0.17	0.09	0.15	***0.04***	***0.01***	***0.01***	***0.00***	***0.01***	0.08–1.4
IgA (g/L)	***0.26***	***0.25***	2.02	1.45	***0.27***	***0.25***	***0.25***	***<0.10***	***0.26***	0.7–4
IgM (g/L)	***0.19***	***0.16***	0.54	0.68	***0.28***	***0.17***	***0.18***	***<0.03***	***0.18***	0.4–2.3
**Proliferation (% of daily control): year**		2018	2018	2018	2015	2018		2017	2016	
PHA	n.a.	***48***	130	135	195	***94***	n.a.	121	115	100
ConA	n.a.	***75***	159	261	134	120	n.a.	n.a.	***95***	100
PWM	n.a.	***28***	244	174	***77***	***48***	n.a.	***63***	***64***	100
PPD	n.a.	***61***	212	***11***	275	***88***	n.a.	n.a.	103	100
IL-2	n.a.	***96***	***83***	***9***	156	161	n.a.	***1***	***33***	100
CD3	n.a.	***70***	124	140	***66***	***74***	n.a.	202	111	100
**Full blood count: year**	2017	2018	2018	2018	2015	2018	2011	2016	2016	
WBC (cells/μl)	8400	4100	8800	7000	6200	6600	7000	7704	9300	3600–11500
Lymphocytes (% leukocytes)	***9***	44	28	33	29	***18***	***20***	27	22	20–44
Lymphocytes (cells/μl)	***756***	1804	2464	2310	1798	1188	1400	2080	2046	1100–4000
**Phenotypic profile of peripheral blood lymphocytes:**										
CD3+ T cells (% lymphocytes)	***32.6***	93.4	62.9	72.9	84.3	80.3	83	79	68.7	55–83
CD4+ T cells (% lymphocytes)	***2.3***	***34.3***	25.7	37.5	64.1	41.4	58	43	50.4	28–57
CD8+ T cells (% lymphocytes)	***9***	64.2	34	35.2	26	35.2	25	33	17.4	10–39
CD19+ B cells (% lymphocytes)	25.3	***0.9***	12.3	6.3	***4.1***	***2.7***	8	8	***5.5***	6–19
CD3+CD56+ NK cells (% lymphocytes)	37.3	***3.6***	11.5	15.7	7.6	***4.4***	8	13	***5.9***	7–31
**Phenotypic profile of peripheral blood CD4+** **T cells:**										
Naive T cells (% CD4+ T cells)	***46***	***8***	51.1	69	84	51	n.a.	***28***	64	49–72
Memory T cells (% CD4+ T cells)	53	91	48.9	***31***	***15***	48	n.a.	71	35	34–71
Recent thymic emigrant T helper cells (% T cells)	30.4	49.9	78.8	83.8	38.9	49.4	n.a.	n.a.	74.8	6.4–42
**Phenotypic profile of peripheral blood CD8+T cells:**										
Early effector memory T cells (% CD8+ T cells)	64.5	15.4	10.4	3.2	9.6	16.8	n.a.	n.a.	3.8	2.9–16
Late effector memory T cells (% CD8+ T cells)	27.5	58.1	4	14.6	51.4	44.1	n.a.	n.a.	21.5	2.6–58
**Phenotypic profile of peripheral blood CD19+** **B cells:**										
Naive B cells (% CD19 B cells)	93.9	n.m.	59.4	77.3	66.6	32.8	n.a.	82.4	91.4	29–93
IgM+ memory B cells (% CD19 B cells)	4.6	n.m.	6.8	6	26.6	9.4	n.a.	***1.4***	4.3	2–25
Class-switched B cells (% CD19 B cells)	***0.5***	n.m.	***2.3***	***1.3***	4.6	9.8	n.a.	***0.4***	***0.2***	3–23
Transitional B cells (% CD19 B cells)	2.7	n.m.	1.9	3.9	4.2	4.7	n.a.	2.1	14.2	0.6–4.6
Plasmablasts (% CD19 B cells)	0.9	n.m.	0.7	2.1	3.7	***0.4***	n.a.	***0.1***	3.9	0.4–3.6
CD21^low^ B cells (% CD19 B cells)	***0.8***	n.m.	2.8	2.2	4	4.3	n.a.	5.1	3.8	1–26

*n.a., not available; n.m., not measurable (due to the extreme low absolute B cell count); WBC, White blood cell; PHA, phytohemagglutinin; ConA, concanavalin A; PWM, pokeweed mitogen; PPD, purified protein derivative; IL-2, interleukin 2. Bold and italic denote values deviating from reference range*.

The first clinical signs were mostly recurrent upper respiratory tract infections (*n* = 6), occurring at a mean age of 36.9 (median age of presentation of first clinical signs: 37 years, age range: 16–50 years). All but two of them developed clinically evident immunodeficiency with recurrent infections (*n* = 6). One patient (S1) presented recurrent episodes of fever and arthritis as first manifestation at the age of 16 years, diagnosed as adult-onset Still's disease. Two mutation carriers presented no significant infection record until time of reporting. All but one individual had hypogammaglobulinemia, which was diagnosed at a mean age of 37.5 years (median age of first diagnosis of hypogammaglobulinemia: 39.5 years, age range: 18–53 years). Infectious manifestations included mostly recurrent upper respiratory tract infections (such as sinusitis, otitis media, bronchitis) and pneumonias (among else due to *S. pneumoniae*) as well as recurrent gastrointestinal infections. Patient S2 was diagnosed with a CMV-colitis, which may have been the consequence of additional immunosuppression due to an—at that time—administered chemotherapy (see below) and patient S6 presented with recurrent warts (*verrucae vulgares*). All seven subjects with recurrent infections and hypogammaglobulinemia were treated with antibody replacement therapy, which led to a substantial reduction in infection frequency, preventing further severe infections such as pneumonias. Besides episodes with fever and arthritis, autoimmune manifestations included idiopathic thrombocytopenic purpura (S7) and autoimmune hemolytic anemia (S1). Other non-infections manifestations included splenomegaly and lymphadenopathy (S1, S2, and S7) as well as nodular lymphoid hyperplasia of the gastrointestinal tract (S2) and nodular regenerative hyperplasia of the liver (S2). Patient S2 was diagnosed with a malignant diffuse large B cell lymphoma at the age of 51, which was treated with R-CHOP chemotherapy. So far, ~10 years after completion of the last chemotherapy cycle, there has been no evidence of lymphoma relapse. Clinical characteristics of all nine studied individuals are summarized in Table 1.

Immunological investigations revealed hypogammaglobulinemia with reduced levels of all three main immunoglobulin classes in seven subjects with recurrent infections. Between the two subjects presenting mild symptoms, one displayed slightly reduced IgG levels and the other had only reduced IgG2-levels with normal values for total IgG and the remaining IgG subclasses. Peripheral blood lymphocyte phenotyping revealed a reduction in absolute CD19^+^ B cell counts in three (S2, S5, and S6) and reduced memory B cells (CD19^+^CD27^+^) in six out of nine studied individuals (S1–S4 and S8, S9). Regarding T cells, most of the studied individuals presented normal or no significantly reduced T cell, CD4^+^ and CD8^+^ T cells. Immunological findings consistent with a late-onset combined immunodeficiency, such as reduced absolute CD4^+^ T cell counts and/or reduced naïve CD4^+^ T cells, have been identified in only two patients (S1 and S2). Lymphocyte proliferation in response to diverse mitogens has been evaluated in a minority of patients revealing inadequate responses to pokeweed mitogen (PWM) and phytohaemagglutinin (PHA) in some patients. Immunological investigations of all nine studied individuals with *NFKB1* mutations are summarized in Table 2.

### Identification of Five *NFKB1* Mutations by Targeted Next-Generation Sequencing in a Cohort of 270 Patients With Antibody Deficiency

Targeted next generation sequencing revealed five likely causative variants in *NFKB1* (Table 3). We analyzed 270 patients with antibody deficiency and identified five monoallelic mutations in *NFKB1* in nine subjects. These include two frameshift mutations due to single base-pair deletions, two frameshift mutations due to single base-pair duplications, and one missense mutation due to a single base pair substitution. All mutations were validated by Sanger sequencing and predicted to be deleterious by *in silico* tools (PolyPhen, Mutation Taster). Except for one variant (c.904dupT, p.S302Ffs^*^7) all the others were not listed in the ExAc database or 1000 genome project.

**Table 3:** Summary of the *NFKB1* variants identified in our cohort.

**Fam ID (Subject ID)**	**Chromosome 4 position**	**Nucleotide change**	**Type**	**cDNA**	**Protein**
Fam A (S1)	103504062	delG	Deletion	c.874delG	p.G292Vfs*140
Fam B (S2-S4)	103504091	dupT	Duplication	c.904dupT	p.S302Ffs*7
Fam C (S5, S6)	103505922	delT	Deletion	c.1012delT	p.S338Lfs*94
Fam D (S7)	103522139	dupA	Duplication	c.1726dupA	p.I567Nfs*6
Fam E (S8, S9)	103498095	G>C	Missense	c.470G>C	p.R157P

### Investigation of Segregation and Clinical Penetrance in Our Cohort

**Figure 1:**
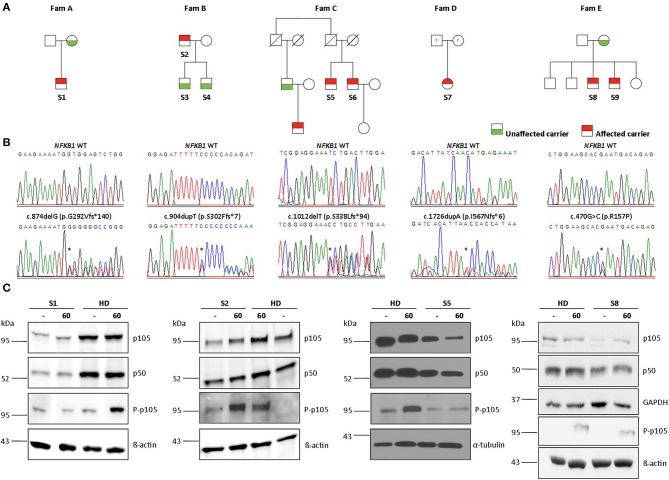
Monoallelic *NFKB1* mutations result in *NFKB1* haploinsufficiency. **(A)** Segregation of *NFKB1* variants were analyzed by sequencing of genomic PCR products and revealed an autosomal-dominant inheritance in families with incomplete clinical penetrance. **(B)** Sequencing of genomic PCR products result in chromatograms of haploinsufficiency mutations, precursor-skipping mutation, and missense mutation of wild type (WT) and affected individuals. **(C)** PBMCs from affected individuals and healthy donors (HD) were stimulated with PMA plus ionomycin for 60 min as indicated. PBMC lysates were analyzed for amounts of p105 and p50 and for phosphorylated p105 (Ser933, P-p105) by Immunoblotting. ß-actin, α-tubulin, and GAPDH were used as loading controls. Individual lanes of WB for S1 and S8 have ben reordered for publishing purposes. Mutations are marked with asterisk.

Four out of 5 families showed evidence of familial disease (Figure 1). We were unable to investigate sporadic or familial occurrence in subject 7, due to the unavailability of samples from relatives. The clinical penetrance of the disease phenotype associated with *NFKB1* variants was incomplete with about 60% in our cohort. We present 13 heterozygous variant carriers, but only eight affected subjects.

### Frameshift Variants Localized in the Rel Homology Domain Lead to p50 Haploinsufficiency

Sequencing of families A, B, and C revealed two single base pair deletions: (1) c.874delG; (2) c.1012delT, and a single base pair duplication c.904dupT (Table 3). These mutations are predicted to cause a shift of the reading frame and a premature termination of translation (Table 3) with a 431 amino acids truncated protein in both families A and C (p.G292Vfs^*^140; p.S338Lfs^*^94) and a 309 amino acids truncated protein for family B (p.S302Ffs^*^7). The latter variant has previously been reported but has not been functional assessed (7, 28). A frequency of 8 ×10–^6^ was found for this mutation in the ExAc database. These three mutations are localized N-terminal in the Rel homology domain (RHD), thus affecting both, p105 and p50. The severely truncated proteins are probably non-functional and undergo rapid decay, therefore leading to p50 haploinsufficiency. To confirm the predicted protein defect of these mutations, we assessed p105 and p50 levels by Western blotting of crude lysates from PBMCs. In all samples derived from affected subjects, expression levels of both, p105 and p50, were reduced to ~50% of the protein levels observed in controls. However, truncated mutant proteins were undetectable on all western blots from families A, B, and C. Phosphorylation of p105 at its C-terminal end in response to stimulation with PMA and ionomycin was tested by Western blotting. Detection of decreased phosphorylation in case of all mutant proteins rather reflects decreased baseline expression levels of p105. Overall, our data suggest that disease phenotype in families A, B, and C results from insufficient p50 levels.

In CMV-promoter-driven overexpression assays using transiently transfected HEK293T cells, EGFP-fused-S338Lfs^*^94 showed an aberrant cytoplasmic localization with accumulation in vacuolic structures in confocal fluorescence microscopy (**Figure 3A**), reminiscent of previously reported damaging mutations. In addition, the mutant protein was detectable by Western blot only after a long exposure time indicating rapid decay (**Figure 3B**).

### Frameshift Variant Localized in the Central Part of p105 Leads to Skipping of the Full Length Precursor

We identified in subject 7 a novel heterozygous single base-pair duplication (c.1726dupA), resulting in a frameshift and a premature termination of translation (Table 3). Bioinformatics analysis predicted a truncated protein product of 572 amino acids (p.I567Nfs^*^6). This mutation affects the first Ankyrin repeat (ANK) and deletes the major part of the ANK domain and the death domain in the C-terminal half of the p105 precursor protein.

Confocal microscopy showed that the fluorescence signal exclusively localizes to the nucleus after forced expression of EGFP-fused-I567Nfs^*^6 in HEK293T cells, suggesting the presence of a p50-like nuclear protein (**Figure 3A**). In contrast, EGFP-fused WT p105 showed a cytoplasmic localization as expected, whereas EGFP-fused WT p50 showed a constitutive nuclear localization in these assays (**Figure 3A**), although under physiological conditions, nuclear translocation of p50 is dependent on IKBα degradation. Western blotting showed a band with the predicted size of ~89 kDa (**Figure 3B**) and a second smaller band (~74 kDa) probably corresponding to EGFP-p50. These observations confirm that the c.1726dupA mutation leads to skipping of the full length precursor, and instead provokes the expression of truncated protein (I567Nfs^*^6) which might be further processed. Therefore, this variant was categorized in the 2^nd^ group of *NFKB1* gene mutations, representing mutations causing skipping of the precursor stage.

### Single Base-Pair Substitution Causes a Reduction of p105

**Figure 2:**
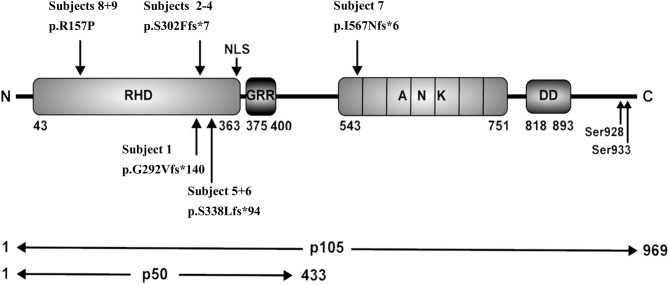
Schematic representation of protein domains and genetic variants adapted from Fliegauf et al. RHD, Rel homology domain; NLS, nuclear localization sequence; GRR, glycine-rich region, ANK, ankyrin repeat domain; DD, death domain.

In family E, two brothers carry the missense mutation c.470G>C (Table 3). This transversion leads to a substitution of arginine for proline at position 157 (p.R157P), located in the N-terminal third of the RHD (Figure 2). This variant can be therefore assigned to the 3^rd^ group of *NFKB1* mutations, affecting both, the precursor p105 and the mature p50. Western blot analysis of PBMCs in both studied carriers of the p.R157P variant revealed reduced levels of both p105 and p50, while the phosphorylation of residually expressed p105 remained intact (Figure 1C and Supplementary Figure 2).

**Figure 3:**
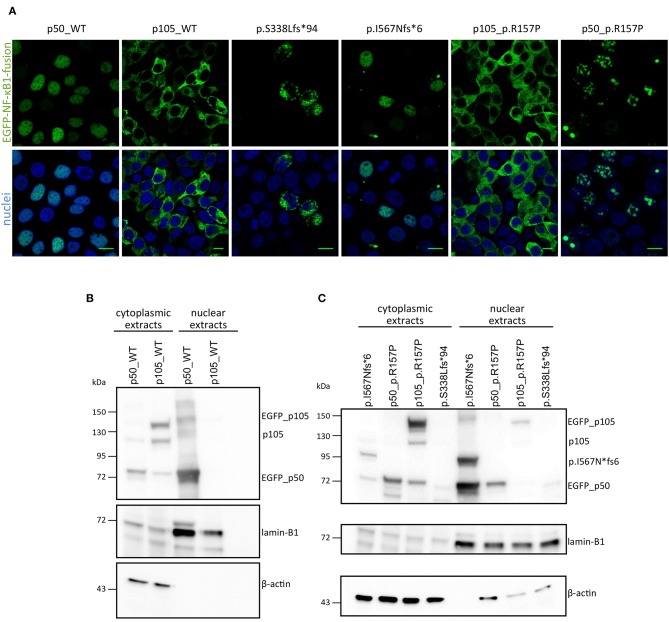
Overexpression assay of EGFP-fused NF-κB1 proteins. **(A)** HEK293T cells were transiently transfected with EGFP-fused mutant proteins or EGFP-fused wildtype proteins. Scale bars represent 10 μm. Immunoblots with in either nuclear or cytoplasmic fraction of transiently transfected HEK293T cells with N-terminally EGFP-tagged p50-WT or p105-WT **(B)** or mutant-constructs (**C**; as indicated) were analyzed for p105 and p50 amounts. ß-actin was used as a cytoplasmic loading control and lamin-B1 for nuclear loading control.

In contrast to the prominent defects of haploinsufficiency and precursor skipping mutations, the missense mutation in Family E caused a milder reduction in of both p105 and p50 protein expression levels in both affected brothers compared to healthy donors (Figure 3C and Supplementary Figure 2). We modeled the p.R157P variant onto the known protein structure of p50 and found that it was located in the central part of a predicted α-helix, though at some distance to the DNA binding site (Supplementary Figure 1). Since a proline residue can interfere with helical protein structures and thus might abrogate protein-protein interaction or the DNA-binding ability of p50, we performed further functional studies. In transfected HEK293T cells the EGFP-fused full length p105-R157P mutant was localized to the cytoplasm and gained expression levels undistinguishable from the wild-type counterpart, as confirmed by confocal microscopy and Western blotting, respectively (Figure 3). Although expressed at levels comparable to the wild-type control, the EGFP-tagged p50 R157P mutant protein displayed an aberrant nuclear localization pattern, with irregular accumulation in aggregates instead of a uniform distribution (Figure 3). Since the highly intense fluorescence signals were apparently co-stained with DAPI, we concluded that the mutant p50 is functionally altered, although it can enter the nucleus.

## Discussion

### *NFKB1* Variants Present the Most Common Monogenic Cause in a Cohort of 270 Patients With Antibody Deficiency

In this study we performed targeted next-generation panel sequencing of 270 patients and identified five monoallelic pathogenic *NFKB1* variants in 5 unrelated families, including four *novel* ones. Thereby, *NFKB1* variants represented the most frequent known monogenic cause in our cohort. *NFKB1* variants segregated in an autosomal-dominant mode with an incomplete clinical penetrance of about 60%. Despite evident phenotypic heterogeneity most of studied subjects (i.e., 8 out of 9) displayed hypogammaglobulinemia. Furthermore, we showed that identified mutations lead to functionally alterations in the NF-κB1 subunits p105 and p50.

### Haploinsufficiency Mutations Lead to Loss-of-Function

In three families (A, B, C) we detected frameshift mutations in the N-terminal half of p105, which are predicted to cause a truncation of protein length due to a premature termination of translation. These truncated proteins might undergo rapid proteasomal degradation or might be non-expressible due to nonsense-mediated mRNA decay (NMD). Thus, the truncated mutant proteins were undetectable on Western blot of patient cells (Figure 1). Typically, haploinsufficiency mutations due to early frameshifts (i.e., affecting the RHD) would lead to expression of severely truncated proteins that lack the nuclear localization sequence (NLS) and therefore stay cytoplasmic where they undergo decay, as observed with the p.S338Lfs^*^94 variant upon ectopic expression in HEK293T cells (Figure 3). Due to heterozygosity, overall expression of both p105 and p50 (derived from the remaining wild type allele) is reduced to ~50% of the levels observed in healthy controls, as has been previous described for various *NFKB1* haploinsufficiency variants (7, 10, 11).

The mutation that we identified in Family B (c.904dupT, p.S302Ffs^*^7) has previously been reported by others in three affected individuals (7, 28). However, functional investigation has not been performed. Rae et al. (28) reported a male individual (P9.1) and his son (P9.2) with predominant antibody deficiency and autoimmune manifestation. The father presented with recurrent upper respiratory tract infections, upper zone lung fibrosis, autoimmune hemolytic anemia (AIHA), and splenomegaly. The 9-years-old son presented with panhypogammaglobulinemia, idiopathic thrombocytopenia purpura (ITP), AIHA, autoimmune neutropenia and mild splenomegaly. Tuijnenburg et al. (7) reported a female patient (N:II-1) with onset manifestation of hypogammaglobulinemia, generalized lymphadenopathy, splenomegaly and pancytopenia. She presented with EBV and CMV infections and neutropenic sepsis. In addition, she showed autoimmune manifestation with *alopecia totalis* and breast cancer. All patients had antibody deficiency and splenomegaly and three out of four suffered from cytopenia, which has also been reported in other studies (11, 22). In addition, our patient (S2) and the patient reported by Tuijnenburg (N:II-1) both had CMV infection and cancer (S2: non-Hodgkin lymphoma; N:II-1: breast cancer). Our patient (S2) showed the most severe phenotype among the affected individuals of our cohort. Notably, both sons, who are both mutation carrier had only mild symptoms at the time of this study. The older son (S3, 33 years) displayed IgG2 subclass deficiency and asthma. The younger son (S4, 24 years) presented with slightly reduced amount of total IgG and also asthma. We therefore recommend a closer follow-up of these subjects, because Ig isotype or IgG subclass deficiencies can develop into CVID over time (29–31). In addition, an age-dependent manifestation of the *NFKB1*-related phenotype has been reported (4).

### Precursor Skipping Variant Leads to Expression of p50-Like Protein

In contrast to haploinsufficiency mutations, frameshift mutations in the central part of p105 (typically on the C-terminal side of the NLS), lead to skipping of the precursor stage and expression of p50-like mutant proteins as previously suggested (23, 32). The mutation (p.I567Nfs^*^6) in Family D (S7) also predicts the expression of a p50-like protein, however with a molecular mass of ~89 kDa (which probably processed to p50) and which retains the NLS and translocates into the nucleus upon expression in HEK293T cells (Figure 3). Tuijnenburg et al. (7) describe a reduced expression of wild-type p50 (which is derived from the non-mutant allel) in all analyzed patient samples with precursor skipping variants and confirmed the presence of a shorter mutant p50-like protein (derived from the mutant allele) in one subject. Due to lack of samples from our patient we were unable to confirm our observations in PBMCs. In contrast to previously described precursor skipping mutations the mutation p.I567Nfs^*^6 is localized in the ankyrin repeat domain (ANK), therefore suggesting an impaired protein–protein interactions, but the precise molecular defect is still unknown.

### Missense Variant Disables Intramolecular Contacts

In family E (S8/S9) we found a missense mutation localized in the N-terminal half of both p105 and p50, leading to a single amino acid change (p.R157P). The conserved arginine on position 157 in almost all available vertebrates at UCSC Genome Browser, suggests an important role of this amino acid for the function of p105 and/or p50. The variant is located in the N-terminal third of the RHD (Figure 2), which mediates DNA motif binding, dimerization and interaction with inhibitory components. Modeling p.R157P on the p50 structure revealed its position in the central part of a predicted α-helix, although the arginine 157 does not appear to be involved in direct DNA contact. However, the model predicted that Arg157 forms polar contacts with Ala111, His112, and Ser113 in a flexible loop region, and these contacts are lost in the Pro157 mutant. Furthermore, since a proline residue can interrupt α-helical protein structures and has been estimated to bend helices by an angle of ~10–20 Å (33, 34), the proline-induced kink in the RHD might disturb structural features likely with consequences for function and/or the interaction with other proteins.

Our Western blot results indicate a slight reduction of p105 in both samples derived from patients with a heterozygous p.R157P mutation, similarly as previously reported for other missense variants. We found the expression and cytoplasmic localization of the mutant p105 undistinguishable from the WT p105, whereas the mutant p50 revealed an aberrant intranuclear pattern compared to the wildtype counterpart, indicating that the functional defect predominantly affects the mature p50. The substitution of arginine for proline destroys intramolecular contacts and may lead to insufficient folding of p50, which might lead to altered protein-protein or DNA binding and eventually to the granular deposition in overexpression studies. In conclusion, although missense mutations might not necessarily lead to a complete loss-of-function the subnuclear mislocalization of the p50-p.R157P mutant indicates a servere defect.

### Alteration in *NFKB1* Causing p50 Haploinsufficiency Associated With Various Clinical Manifestations

Mutations in *NFKB1* causing haploinsufficiency of p50 can lead to intrinsic B cell disorders and therefore to a new subgroup of CVID (CVID12, OMIM# 616576) characterized by recurrent infections with hypogammaglobulinemia (11). In the last 4 years it was shown that *NFKB1* mutations cause a wider spectrum of disease manifestations, including combined B- and T-cell dysfunction, autoinflammation (24), and hyperinflammatory reactions and Epstein-Barr virus (EBV) (8) infections (30, 31). In our cohort, the clinical severity was highly variable, similarly as also reported by others (10). The most common clinical manifestations were hypogammaglobulinemia, lymphoproliferation especially splenomegaly and autoimmune features like cytopenia.

All carriers of *NFKB1* mutations showed reduced number of switched memory B cells and a broad range of non-switched memory B cells. A classification of affected carriers and non-affected carriers by an increased number (over 10%) of CD21^low^ B cells (7), was not applicable in our cohort due to decreased numbers in all subjects. In contrast, an increased number of CD21^low^ B cells might be a direct consequence of defective *NFKB1* function (35–37).

In conclusion, we report on nine subjects bearing five damaging monoallelic mutations in *NFKB1* leading to haploinsufficiency of p50. *NFKB1* variants presented the most frequent known monogenic cause in our cohort and segregated with immunodeficiency in the families, though with an incomplete penetrance of the clinical manifestation.

## Data Availability Statement

All datasets analyzed for this study are included in the article/Supplementary Material.

## Ethics Statement

The study was approved by the institutional medical ethical committee at Hannover Medical School (ethics approval number: 5582). The written consent of all study participants was obtained. Informed consent was obtained from patients and patients’ family members, respectively, according to ethical and legal guidelines.

## Author Contributions

Research design: RS, FA, BG, and CSchr. Sample collection: FA, CSchr, GS, and LH. Targeted resequencing and NGS data analysis: FA and CSchr. Performance of functional experiments and data analysis: CSchr, MF, RJ, TD, DL, CSche, and SS. Writing and contributing to writing of the manuscript: all authors.

### Conflict of Interest

The authors declare that the research was conducted in the absence of any commercial or financial relationships that could be construed as a potential conflict of interest.

## Supplementary Material

Supplementary Figure 1 Missense mutation R157P enables intramolecular contacts. **(A)** Structural model of p50 protein (green) with DNA (backbone orange, basepairs blue). Arginine residues are labeled in pink and the position of Arg157 is indicated by an arrow. **(B)** Detailed view of the α-helix with either wildtype (arginine, left) or mutant (proline, right) residues. Note that Arg157 enables intramolecular polar contacts (yellow dotted lines) with Ala111, His112, and Ser113 that are lost in the Pro157 mutant.

Supplementary Figure 2 Densitometric analysis of p105 and p50 expression show decreased expression of p105 and p50. PBMCs from affected individuals and healthy donors (HD) were stimulated with PMA plus ionomycin. PBMC lysates were analyzed for amounts of p105, p50 and phosphorylated p105 (P-p105) by Immunoblotting shown in Figure 1C. **(A)** Densitometric analysis based on WB from PBMCs of S1, revealing decreased expression of p105 and p50 and inadequate phosphorylation of P-p105, as compared to a HD. **(B)** Based on densitometric analysis of WB from PBMCs of S2, haploinsufficiency mutation revealing decreased expression of p105 and p50 and adequate phosphorylation of P-p105, as compared to a HD. **(C)** Densitometric analysis based on WB from PBMCs of S5, revealing decreased expression of p105 and p50 and inadequate phosphorylation of P-p105, as compared to a HD. **(D)** Missense mutation c.470G>C lead to reduced expression of p105 and p50 and adequate phosphorylation of P-p105, as compared to a HD, based on densitometric analysis of WB from PBMCs of S8. **(E)** PBMCs from affected individual S9 and healthy donor were stimulated with PMA plus ionomycin for 60 min as indicated. PBMC lysates were analyzed for amounts of p105 and p50 by Immunoblotting. ß-actin was used as loading control. **(F)** Densitometric analysis of p105 and p50 expression, based on WB from **(E)** showing decreased expression of p105 and p50 in S9 as compared to a HD.

Supplementary Table 1 Genes included in the NGS panel.

Supplementary Table 2 Primers used for Sanger sequencing.

## Abbreviations

CID: combined immunodeficiency
CVID, common variable immunodeficiency, *NFKB1*: nuclear factor kappa B subunit 1
*NFKB2*: nuclear factor kappa B subunit 2.
