# Supplementary figures and images for: Late-Onset Antibody Deficiency Due to Monoallelic Alterations in NFKB1
Source: Front Immunol. 2019 Nov 14;10:2618. doi: 10.3389/fimmu.2019.02618 (PMC6871540; doi:10.3389/fimmu.2019.02618)

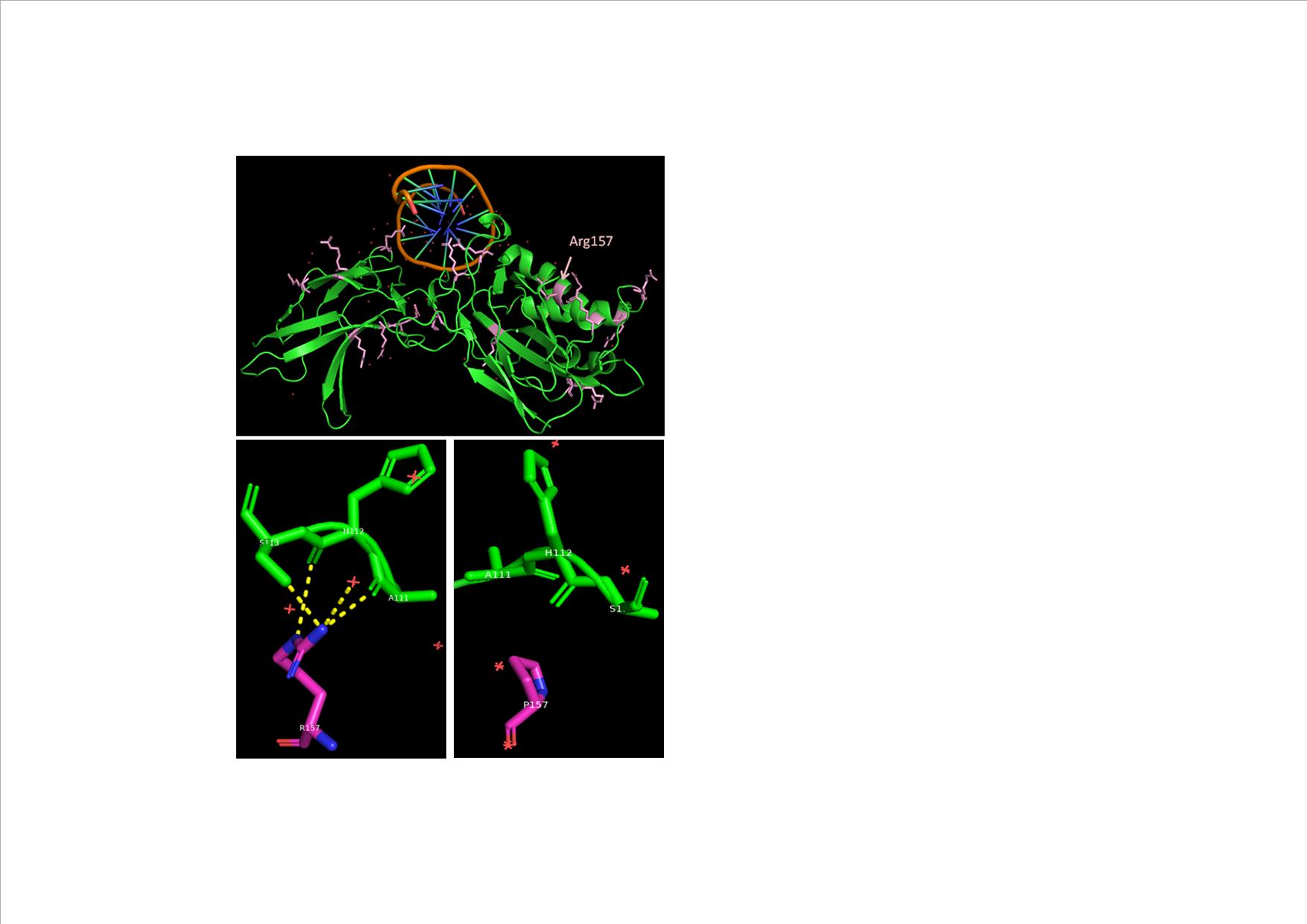

Supplement: Supplementary Figure 1 — Missense mutation R157P enables intramolecular contacts. (A) Structural model of p50 protein (green) with DNA (backbone orange, basepairs blue). Arginine residues are labeled in pink and the position of Arg157 is indicated by an arrow. (B) Detailed view of the α-helix with either wildtype (arginine, left) or mutant (proline, right) residues. Note that Arg157 enables intramolecular polar contacts (yellow dotted lines) with Ala111, His112, and Ser113 that are lost in the Pro157 mutant. [file Image_1.JPEG]

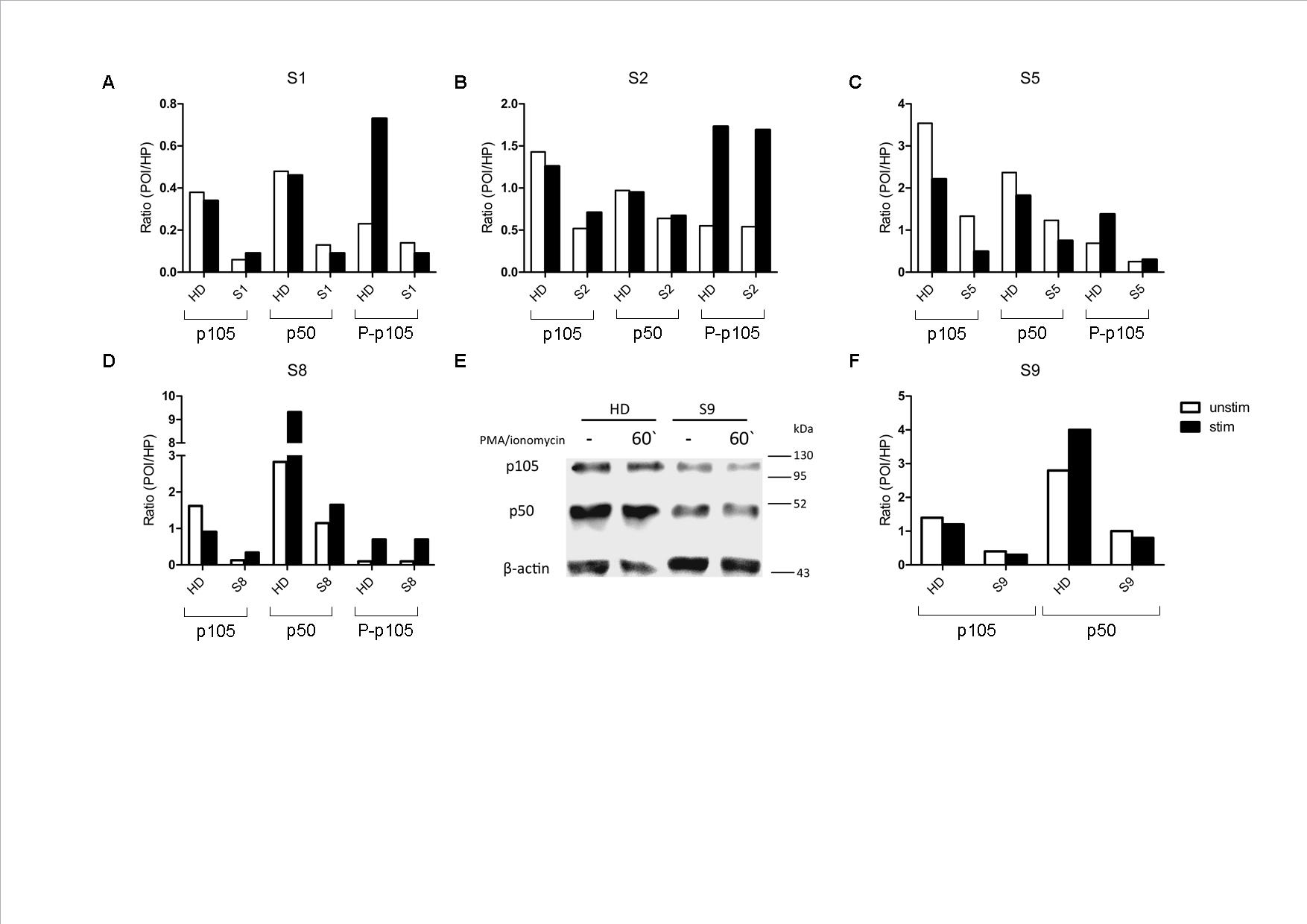

Supplement: Supplementary Figure 2 — Densitometric analysis of p105 and p50 expression show decreased expression of p105 and p50. PBMCs from affected individuals and healthy donors (HD) were stimulated with PMA plus ionomycin. PBMC lysates were analyzed for amounts of p105, p50 and phosphorylated p105 (P-p105) by Immunoblotting shown in Figure 1C. (A) Densitometric analysis based on WB from PBMCs of S1, revealing decreased expression of p105 and p50 and inadequate phosphorylation of P-p105, as compared to a HD. (B) Based on densitometric analysis of WB from PBMCs of S2, haploinsufficiency mutation revealing decreased expression of p105 and p50 and adequate phosphorylation of P-p105, as compared to a HD. (C) Densitometric analysis based on WB from PBMCs of S5, revealing decreased expression of p105 and p50 and inadequate phosphorylation of P-p105, as compared to a HD. (D) Missense mutation c.470G>C lead to reduced expression of p105 and p50 and adequate phosphorylation of P-p105, as compared to a HD, based on densitometric analysis of WB from PBMCs of S8. (E) PBMCs from affected individual S9 and healthy donor were stimulated with PMA plus ionomycin for 60 min as indicated. PBMC lysates were analyzed for amounts of p105 and p50 by Immunoblotting. ß-actin was used as loading control. (F) Densitometric analysis of p105 and p50 expression, based on WB from (E) showing decreased expression of p105 and p50 in S9 as compared to a HD. [file Image_2.JPEG]
